# Surgical Treatment of Diseases of Metabolism

**Published:** 1914-02-14

**Authors:** 


### SURGICAL TREATMENT OF DISEASES OF METABOLISM.

The enormous strides which modern surgery has made during the last few years is nowhere better illustrated, perhaps, than in the recognition which has been accorded to the surgical treatment of certain diseases of metabolism which hitherto have been regarded as quite outside the bounds of legitimate surgical interference. A recent article by Professor Borckenheimer, of Berlin, contributed to the post-graduate journal *Zeitschrift für Aerztliche Fortbildung*, gives an interesting *résumé* of what this treatment has been able to accomplish. Borckenheimer differentiates sharply between two classes of surgical interference; those, first, in which the operation needed is in order to correct a lesion produced by the disease, and those which have for their object the disease itself—either its cure, if possible, or its amelioration in some tangible degree. In every case he counsels the close co-operation between physician and surgeon without which both patient and surgeon expose themselves to grave risk. An operation on a patient who is suffering from a severe constitutional disease, such as diabetes, is attended with far more serious risk than one upon a patient who is not the subject of metabolic disorder. In such circumstances the surgeon ought to approach the case with the greatest circumspection A careful and methodical examination of all the systems should be a necessary preliminary to any interference. Further, the utmost attention should be given to those little essentials of surgical cleanliness, both at the operation itself and during the after-treatment of the case, which conduce so much to ultimate success. Still another serious question is the choice of an anæsthetic. Sufferers from diabetes and obesity do not stand general anæsthesia well; the latter, again, are not ordinarily proper subjects for purely local anæsthesia. Probably the best method in these cases is to use combined anæsthesia, such as scopolamine, morphine, ether, omnopon, and gas, or ether, oxygen, and a narcotic. Whether any special precautions must be taken in addition will largely depend on the case itself. Where the surgeon fears infection, for various reasons, the question of a prophylactic administration of some anti-bacterial serum will have to be considered. So far surgical interference in diseases of metabolism has been limited to diabetes, obesity, and gout.

### The Surgery of Diabetes.

Diabetic patients are well known as extremely bad subjects for operation. In every case where surgical interference is necessary the patients ought to be carefully prepared by dieting and methodical cleansing of the skin. At the operation chloroform should be avoided and ether or combined narcosis substituted. In the dressing of the wounds certain antiseptics should similarly be avoided; instead of using iodoform gauze Borckenheimer advises the use of a camphor solution in alcohol or some other liquid antiseptic dressing. In the after-treatment diabetic diet must be rigorously prescribed, the patient carefully nursed and his skin kept sweet, and his strength must be maintained by every possible means. Insomnia is frequently complained of, and must be combated with the liberal use of narcotics of the opium group.

In true diabetes, as distinguished from glycosuria, surgery has so far been impotent to afford much relief from the disease itself. It is true operations on the pancreas and thyroid have been suggested, and in one or two cases carried out, but the results have not been encouraging. On the other hand, surgery has been able to afford considerable relief to patients afflicted with lesions which have been directly the result of their disease. Of these diabetic gangrene is the chief. The modern rule is to amputate well above the gangrenous portion; thus gangrene of the foot demands amputation above the knee. Unless such radical interference is instituted there is a risk of spreading cellulitis, which is rapidly fatal. A grave complication in such cases is diabetic coma; against this the injection of soda solution, with ether and camphor as cardiac stimulants, is practically the only remedy. Borckenheimer advises the injection of 750 grains of sodium bicarbonate per day subcutaneously, and states that he has several times succeeded with these drastic measures in saving patients who have been quite unconscious. When the amount of sugar in the urine rises rapidly after an operation on a diabetic patient it is a very grave prognostic sign, and immediate steps should be taken to try and decrease the amount of sugar. In such cases Borckenheimer strongly urges the employment of saline, both per rectum and subcutaneously.

### Surgical Interference in Gout.

Surgical interference in gouty patients is a serious matter, since many of these patients suffer from granular atrophy of the kidneys, while others, again, suffer from glycosuria and obesity. They are in nearly every instance bad subjects for operation. Where an operation is imperatively necessary, the patient ought to be carefully prepared for it. Borckenheimer pins his faith to hydrochloric acid as a preliminary, which he administered in minim doses to the extent of five minims per day, and also advises the administration of tonics, quinine, iron, and arsenic. Special care is to be taken in avoiding incisions into parts affected by the gout: no good results from attempts to remove the tophi by operation. In the after-treatment of operative wounds in gouty subjects obstinate fistulæ are often a serious difficulty, while phlebitis and cellulitis are also frequently met with. For the first Borckenheimer advises treatment by the *x*-rays, Beck's bismuth paste, and Merck's leucofermentin. When a serious bursitis is present the whole bursal sac has to be removed; this should be carefully done under the most rigid aseptic precautions. In conclusion he warns against the use of ice compresses and massage, "since the former increases the gouty attacks, while the latter has a tendency to induce local necrosis, which leads to fistulæ and ulceration."

